# Heart Rate Kinetics and Sympatho-Vagal Balance Accompanying a Maximal Sprint Test

**DOI:** 10.3389/fpsyg.2019.02950

**Published:** 2020-01-22

**Authors:** Jorge L. Storniolo, Roberto Esposti, Paolo Cavallari

**Affiliations:** Human Physiology Section, Department of Pathophysiology and Transplantation, University of Milan, Milan, Italy

**Keywords:** R-R variability, heart rate recovery, maximal exercise, autonomic nervous system, human

## Abstract

When a maximal sprint starts, heart rate (HR) quickly increases. After the exercise ends, HR keeps high for seconds before recovering with a roughly exponential decay. Such decay and its time constant (τ_off_) have been widely studied, but less attention was devoted to the time delay (t_delay_) between sprint end and HR decay onset. Considering the correlation between sympatho-vagal balance and performance, as well as the occurrence of heart failure in cardiopaths during the post-exercise phase, we evaluated sympatho-vagal balance before and after sprint, trying to correlate it with both t_delay_ and τ_off_. R-R intervals, recorded in 24 healthy adults from 5 min before to 5 min after a 60-m sprint-test (from Storniolo et al., 2017, with permission of all authors), were re-processed to extract HR variability power (LF and HF) in the low- and high-frequency ranges, respectively. The sympatho-vagal balance, evaluated in pre-test resting period (LF/HF)_REST_ and at steady-state recovery (LF/HF)_RECOV_, was correlated with t_delay_ and τ_off_. Both (LF/HF)_REST_ and (LF/HF)_RECOV_ had a skewed distribution. Significant rank correlation was found for (LF/HF)_REST_ vs. τ_off_ and for (LF/HF)_RECOV_ vs. both τ_off_ and t_delay_. The difference (LF/HF)_RECOV–REST_ had a normal distribution and a strong partial correlation with t_delay_ but not with τ_off_. Thus, a long t_delay_ marks a sympathetic activity that keeps high after exercise, while a high sympathetic activity before sprint leads to a slow recovery (high τ_off_), seemingly accompanying a poor performance.

## Introduction

Current studies have widely reported the role of heart rate (HR) kinetics after exercise in predicting health issues such as cardiac disease, heart failure, and sudden death (Imai et al., 1994; Aeschbacher et al., 2016; Qiu et al., 2017). It is well known (Task Force of the European Society of Cardiology the North American Society of Pacing Electrophysiology, 1996) that the analysis of HR variability (HRV) needs a steady-state HR kinetic, or at least a linear trend of the signal, in order to reliably calculate variability indices like the SD of R-R intervals and the root mean square of successive differences (in the time domain), or the spectral components of HRV (in the frequency domain).

Concurrently, the specific analysis of the HR decay in the recovery phase from exercise has been as well accepted as a marker of the sympatho-vagal balance (Dimkpa, 2009; Billman, 2011). The advantage of this approach, compared to traditional HRV analysis consists of the possibility of calculating these parameters directly from HR time course (Peçanha et al., 2014; Watson et al., 2017). However, it should be taken into account that some studies reported controversial results when the analysis was restricted to a short part of the decay (commonly no more than 60 s), due to the non-linearity of the signal (Michael et al., 2017).

HRV is acknowledged as an indicator of the cardiac sympathetic and parasympathetic control (Task Force of the European Society of Cardiology the North American Society of Pacing Electrophysiology, 1996). In particular, the fraction of the total power in the low-frequency range (LF, 0.04–0.15 Hz) reflects the sympathetic drive, while the fraction in the high-frequency range (HF, 0.15–0.4 Hz) unveils the parasympathetic drive through the *vagus* nerve. Accordingly, the LF/HF ratio quantifies the sympatho-vagal balance (Malliani et al., 1991). This balance has been shown to undergo significant changes at exercise transitions (basal to exercise to recovery) where its effects may be appreciated by rapid changes in HR (Michael et al., 2017). Fast feedbacks from muscle mechanoreceptors contribute to initial parasympathetic withdrawal as well as to its reverse process upon the exercise cessation (Pierpont et al., 2000; Dupuy et al., 2012). However, the speed of the process may vary in response to different factors, such as the exercise type and intensity, as well as the posture adopted in the baseline and recovery periods (Coote, 2010). It is also worth noting that a low LF/HF ratio, reflecting a generally high vagal tone, has been shown to be associated with a better cardiac adaptation to daily life exercise or activities (Peçanha et al., 2014).

HRV tests do not require expensive apparatuses, besides reliable HR monitors capable of beat-by-beat acquisition. However, its evaluation in exercise protocols commonly needs at least 25 min, spanned among baseline, exercise, and recovery acquisitions (Storniolo et al., 2017). Indeed, a quite long exercise is required to reach a stationary HR, from which HRV frequency distribution is calculated. However, it has been recently shown (Storniolo et al., 2017) that the maximal oxygen consumption, which correlates to the individual’s adaptation to strenuous exercise, may be predicted also by measuring the HR decay after a 60-m sprint exercise, which is so short that the metabolic rate could not even approach the steady-state value.

In this perspective, we reprocessed HR data before, during, and after such sprint test, seeking for parameters of post-exercise HR kinetics that could correlate with sympatho-vagal balance, assessed through HRV frequency analysis in the pre-exercise and full-recovery periods. Should our search be fruitful, an important index could be easily derived from HR monitor data to estimate the individual adaptation to everyday life exercises. Among the HR recovery parameters, we considered the constant of the mono-exponential decay (τ_off_), which is considered an indicator of higher aerobic and anaerobic power as estimated from different running protocols (Otsuki et al., 2007; Borresen and Lambert, 2008; Storniolo et al., 2017). Besides, we evaluated the period of time (t_delay_) for which the HR plateaus between the exercise cessation and the onset of the exponential decay. Such delay in HR decrease has been observed in different physiological markers after exercises, including blood lactic acid concentration and oxygen consumption (Margaria, 1976; Bailey et al., 2018). Actually, t_delay_ in HR recovery has been repeatedly observed in the literature; nevertheless, many authors simply discarded it as a “confounding factor” for the kinetics analysis of HR exponential decay (Buchheit et al., 2007; Peçanha et al., 2014).

It is noteworthy to emphasize that t_delay_ is particularly evident after strenuous exercise, but to our knowledge, it was never correlated to sympatho-vagal balance, or discussed in physiological terms. Thus, given the proven correlation between the sympatho-vagal balance and the exercise performance (Michael et al., 2017), we evaluated HRV before and after a maximal sprint test, trying to correlate the sympatho-vagal balance with both t_delay_ and τ_off_. Our hypothesis states that these parameters (particularly t_delay_) might be associated with the sympatho-vagal balance since parasympathetic reactivation is notably impaired after high exercise intensity.

## Materials And Methods

Heart rate data analyzed in this study were originally collected by Storniolo et al. (2017), who also extracted the onset of the HR mono-exponential decay after exercise and its time-constant τ_off_. Here HR data were re-processed, with kind permission of all co-authors, so as to measure HRV in pre-exercise and full-recovery periods, and correlate it with the time interval between the exercise end and the onset of HR decay, here called t_delay_. Details of population parameters and data acquisition are thus reported in brief (for all details, see Storniolo et al., 2017).

### Subjects

Out of the 25 subjects who attended the Storniolo et al. (2017) study, we excluded data from one subject whose t_delay_ was larger than the population mean + 2 SD. The resulting sample was composed of 24 subjects (7 women and 17 men, 25.0 ± 5.0 years, 1.77 ± 0.08 m height, 71.9 ± 8.5 kg body mass; mean ± SD). They were physically active, being involved either in recreational activity, or in amateur sport activity with a maximum of three sessions per week.

### Experimental Protocol

Subjects performed a 60-m maximal sprint accomplished on an outdoor athletic track. They were instructed to get at the experimental session in a relaxed and fully hydrated state. In addition, they were told to avoid alcohol (24 h) and caffeine (6 h) intake before the test. Subjects were asked to perform the sprint at their best.

### Data Acquisition

The 60-m maximal sprint trial was preceded by a short warm-up (5 min with jogging and stretching) and 10 min of resting period, 5 min in a seated and 5 min in a standing position. HR was recorded beat-by-beat throughout the 5 min of standing rest, the running phase and the 5 min of standing recovery, using a HR monitor with transmitter belt (Polar S410, Kempele, Finland). All tests were performed at the same time of the day (10–11 am) to limit the influences of circadian rhythm on muscle performance and HR response/variability (Task Force of the European Society of Cardiology the North American Society of Pacing Electrophysiology, 1996).

### Sympatho-Vagal Balance

HRV was separately evaluated into the first 3 min of the standing rest and in the last 3 min of the recovery phases. R-R intervals were pre-processed so as to exclude those falling outside ± 2 SD with respect of the mean R-R duration, then the R-R time sequence was resampled at 10 Hz with spline interpolation. The RMS Power Spectral Density was extracted by the Least-Squares autoregressive method, setting the order parameter to the value that minimized the Akaike information criterion (Task Force of the European Society of Cardiology the North American Society of Pacing Electrophysiology, 1996).

The fractions of the total power in the LF and HF bands (LF: 0.04–0.15 Hz and HF: 0.15–0.40 Hz, approximately) were evaluated as the area under the respective spectral peaks, identified by means of the valleys between them. Then the ratio of LF/HF power was calculated before the sprint [(LF/HF)_REST_] and at late recovery [(LF/HF)_RECOV_], so as to evaluate the sympatho-vagal balance in the two phases (Malliani et al., 1991); the higher the ratio, the higher the sympathetic drive. The difference (LF/HF)_RECOV–REST_ was also determined.

## Statistics

Shapiro-Wilk test was used to assess the normal distribution of the data. Linear associations between (LF/HF)_RECOV–REST_ with t_delay_ and τ_off_ were evaluated by Pearson partial correlation coefficients. Spearman’s rank-order correlation was instead applied for (LF/HF)_REST_ and (LF/HF)_RECOV_ because their distribution deviated from normality. Statistical significance was granted at *p* < 0.05.

## Results

**FIGURE 1:**
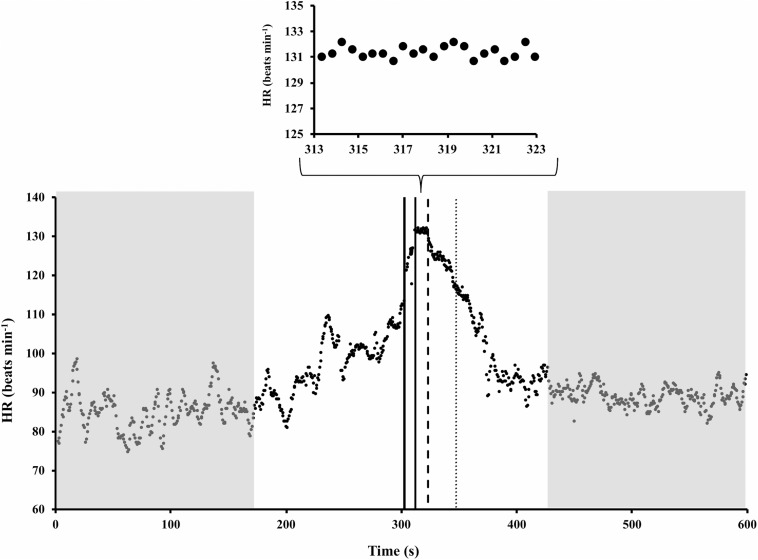
Example of heart rate (HR) time course during standing rest, 60-m sprint, and standing recovery (redrawn from Storniolo et al., 2017). Shadow gray areas represent the time periods for HR variability analysis. It is apparent that in those periods HR was stationary. Solid vertical lines mark the start and end of the sprint. Dashed line marks the onset of HR exponential decay; thus, its distance from the last solid line represents t_delay_. Dotted line is set at the time in which the HR decay was 63% completed, so that its distance from dashed line represents τ_off_.

An example of HR time course during standing rest, 60-m sprint, and standing recovery is illustrated in Figure 1. It is apparent that HR started to rise in advance of the 60-m sprint, as the subject was readying itself to the effort. It is also apparent that after the end of the sprint, it took more than 1 min for the HR to regain a steady state. Thus, the last 2 min of standing rest and the first 2 min after sprint end were excluded from HRV analysis. Note that the HR exponential decay started about 10 s later than the end of the sprint (t_delay_), a value that covered about 1/3 of the decay time-constant (τ_off_). In the experimental sample, t_delay_ resulted to be 8.50 ± 1.98 s (mean ± SD), while τ_off_ was 27.85 ± 6.83 s. The 60-m sprint lasted 9.76 ± 1.37 s.

**FIGURE 2:**
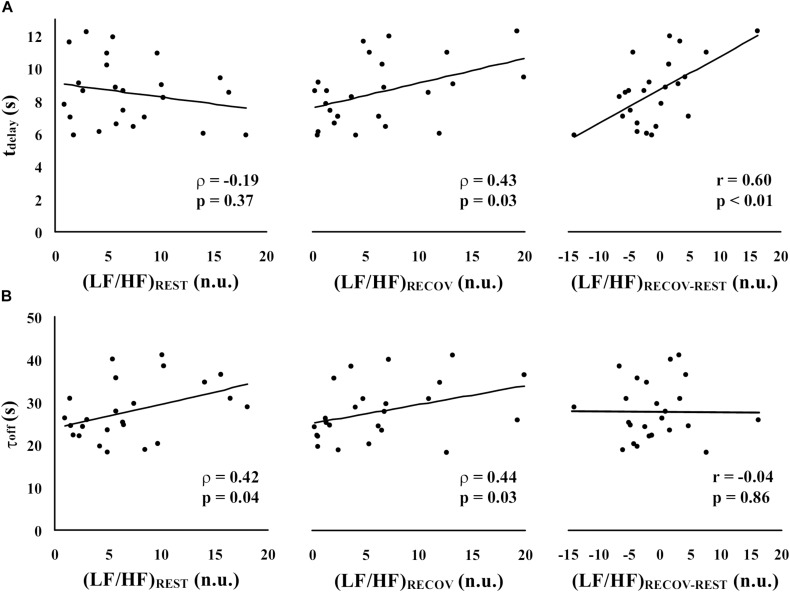
Spearman’s rank correlations (ρ) and Pearson’s partial correlations (r) between each of the HRV-based indicators of sympatho-vagal balance [(LF/HF)_REST_, (LF/HF)_RECOV_, and (LF/HF)_RECOV–REST_] and the parameters characterizing the time-course of HR recovery [t_delay_ in panel **(A)**, and τ_off_ in panel **(B)**].

Figure 2 illustrates, in the experimental sample, the correlations between each of the HRV-based indicators of sympatho-vagal balance [(LF/HF)_REST_, (LF/HF)_RECOV_, and (LF/HF)_RECOV–REST_] and the two parameters characterizing of HR recovery (t_delay_ in panel A, and τ_off_ in panel B). Since (LF/HF)_REST_ and (LF/HF)_RECOV_ significantly deviated from the normal distribution, Spearman’s rank correlations (ρ) were calculated for these variables. Pearson’s partial correlations (r) was instead used for (LF/HF)_RECOV–REST_. A significant positive correlation between (LF/HF)_REST_ and τ_off_ (ρ = 0.42, *p* = 0.043) was found, but not with t_delay_ (ρ = −0.19, *p* = 0.37); note, however, that this last correlation had a negative trend. Instead, (LF/HF)_RECOV_ had a significant positive correlation with both t_delay_ (ρ = 0.43, *p* = 0.035) and τ_off_ (ρ = 0.44, *p* = 0.030). Therefore, while τ_off_ seems more linked to the sympatho-vagal balance in the rest and recovery time periods separately, t_delay_ seems to be a better indicator of the change in sympatho-vagal balance between these two periods. Indeed, the opposite ρ that (LF/HF)_RECOV_ and (LF/HF)_REST_ had with such delay resulted in a strong Pearson’s partial correlation between the difference (LF/HF)_RECOV–REST_ and t_delay_ (*r* = 0.60, *p* = 0.002), while the partial correlation with τ_off_ was at all marginal (*r* = −0.038, *p* = 0.86; Figures 2A,B).

## Discussion

In this study, we aimed to highlight the importance of t_delay_, a representative parameter of the post-exercise HR recovery, as an important variable to assess the sympatho-vagal balance in that phase. In this aim, two time-domain parameters of the fast HR recovery after a maximal sprint-test (t_delay_ and τ_off_) were tested for correlation with the HRV LF/HF ratio, at baseline before the sprint and at late recovery. To our knowledge, the present study is the first that correlates t_delay_ to sympatho-vagal balance and discusses it in physiological terms.

It was found that t_delay_ is a good candidate for indicating the changes in sympatho-vagal balance between resting baseline and late recovery, while τ_off_ seems more linked to the sympatho-vagal balance in each period separately. Indeed, τ_off_ was positively correlated with the sympatho-vagal balance both at rest and at late recovery; thus, it cannot be used to discriminate whether (i) the subject has an overall prevalence for the sympathetic vs. parasympathetic drive, or (ii) he strongly increases his sympathetic drive only after the exercise; in fact, τ_off_ would be high in both cases. On the contrary, the strong partial correlation between t_delay_ and (LF/HF)_RECOV–REST_ clearly witnesses that t_delay_ selectively identifies a subject with problems in recovering his sympatho-vagal balance after a strenuous exercise.

Although t_delay_ has been frequently reported in the literature, it has been often dismissed from analysis due to the difficulty in inquiring the specific chemical dynamics involved during this period (Zhang et al., 2014). Besides that, the persistence of an high HR immediately after the end of an intense exercise might also depend on the high respiratory frequency (Pierpont et al., 2000; Borresen and Lambert, 2008). In accordance with Pierpont et al. (2000), a sustained tachycardia influenced by a high respiratory frequency may indicate a sympathetic predominance, despite the known re-activation of the parasympathetic system, during the fast phase of recovery. Even though some authors (Buchheit et al., 2007; Peçanha et al., 2014) excluded the very first seconds before the start of HR decay, so as to pinpoint the mono-exponential trend, such exclusion may hide part of the autonomic regulation of HR. Considering that a delayed HR recovery has been associated with the healthy and training status of the subject (Borresen and Lambert, 2008), it is of interest to understand whether this delay is secondary to a delayed HR decay (long t_delay_) and/or a slower exponential phase (long τ_off_).

Moreover, it should be noted that “t_delay_” after strenuous exercise has been previously reported not only for HR but also for other physiological markers, such as the lactic acid concentration in the blood, the oxygen uptake and the ventilation (Di Prampero et al., 1973; Margaria, 1976; Bailey et al., 2018). In fact, it is well known that during the exercise recovery phase, the muscle has to pay for the oxygen debt contracted at the beginning. When the load is kept below the maximum aerobic power, the oxygen debt is limited to that needed for phosphocreatine resynthesis, and the oxygen uptake (and the correlated variables) starts to decay as soon as the exercise ends. Instead, when the anaerobic threshold is crossed, also the lactic acid production should be added; therefore, the oxygen uptake should keep high for some seconds, so as to pay the lactacid debt. In this case, the oxygen uptake (and the correlated variables) shows a delayed exponential decay, which may be seen as a clipped exponential curve. Such curve would theoretically decrease immediately after the exercise, starting from the oxygen energetic equivalent of the load, but actually, it is clipped at the maximum oxygen uptake allowed by the cardiopulmonary limits. Acknowledging the correlation between the kinetics of oxygen uptake and HR, the statement mentioned above might explain the HR persistence after intense exercise. Considering that (i) the cardiac output is the product of stroke volume and HR, and that (ii) in exercises exceeding ∼60% of individual’s maximum HR the stroke volume stops raising, it follows that HR remains the only responsible for covering the need of a high perfusion after strenuous exercise (Zhang et al., 2014). In this perspective, our sprint exercise apparently crossed the anaerobic threshold; thus, no wonder that we could observe the clipping in HR recovery. It is then clear that the persistence of a high HR should be associated with a high sympathetic drive, influenced by the “payment” of the lactacid debt.

Notably, the delay in oxygen uptake recovery has been shown to be prolonged in heart failure individuals (Bailey et al., 2018). It is also known that a slow HR decay after exercise can be a good predictor of sudden deaths correlated to heart diseases (Huang et al., 2013; Bailey et al., 2018). In this regard, Huang et al. (2013) showed that in elderly, in women, and in patients with normal chronotropic response during exercise, the slower the return of HR to baseline values, the higher is the probability of a vagal deficit. As justified in section “Introduction,” our protocol may ensure the identification of potential risks in individuals attending the sprint test, with the advantage of a short-time requirement (approximately 10 min) and of the easiness of the measuring apparatus.

A short comment deserves the heterogeneous composition of the tested sample. Actually, we spanned on purpose different ages, training statuses, and days within the infradian cycle of interest (menstruation in women, see Schmalenberger et al., 2019, and testosterone seasonal secretion changes in men, see Smith et al., 2013). Indeed, all these aspects affect both HRV and exercise performance, therefore contributing to enhancing the inter-subject variability. In this way we effectively strengthened the reliability of the observed correlations.

A final note deserves the linkage between sympatho-vagal balance and training/emotional status. Our results also highlighted a predominance of the sympathetic drive [(LF/HF)_REST_ > 1, average HR ∼81 bpm] during pre-exercise rest, although we excluded the elevated HR values that preceded the sprint, thus limiting HRV analysis to the first 3 min of rest. Such result may come from the fact that subjects were resting in the orthostatic position, which promotes a higher sympathetic activation even in no exercise situations (Buchheit et al., 2009). However, this could also be linked to the emotional status related to mind-body preparation for the sprint (Choi et al., 2017). Indeed, it has been recognized how emotional factors, such as anxiety, can influence the sympatho-vagal balance mainly during stress situations (ChuDuc et al., 2013). From this perspective, Sanchéz-Conde et al. (2019) reported a high sympathetic drive in nurse students before their first clinical activities, associated with high subjective stress responses. The reason for such a behavior, as discussed by those authors, was the lack of habituation to the emotional stress of the work. Indeed, more experience could bring more adaptation, like in high-level sports athletes who show a lower sympathetic drive despite the stress situation preceding the competitions. Finally, such sympathetic predominance among our participants could also represent their sub-optimal fitness status. Indeed, the positive correlation we found between (LF/HF)_REST_ and τ_off_ is in agreement with Danieli et al. (2014), who showed that the higher vagal predominance at rest in athletes vs. control subjects correlates with a faster HR recovery after sub-maximal exercise. In this regard, it was also reported that τ_off_ correlates with maximal aerobic capacity (Boullosa et al., 2014; Storniolo et al., 2017), a “gold standard” for assessing fitness level. As a whole, these observations stress the importance of a good preparation, both in terms of fitness and in terms of emotion control, to attain a fast recovery after strenuous exercise. Besides an appropriate physical training, it has been reported that mind activities, as yoga and meditation, drive improvements in parasympathetic reactivation through slow breathing techniques, without requiring the same energy expenditure of physical exercises (Adhana et al., 2013).

## Conclusion

These results confirm our hypothesis that t_delay_ is a significant marker for the autonomic nervous system recovery after a sprint test. Still, further studies are needed to elucidate whether a diverse range of running intensities may influence the t_delay_ and τ_off_, as well as the autonomic cardiac control.

## Data Availability Statement

Data analyzed in this study were obtained from Storniolo et al. (2017) and reprocessed with permission of all authors.

## Author Contributions

PC conceived the study. JS collected the data. JS, RE, and PC analyzed the data, drafted the manuscript, and revised the final version.

## Conflict of Interest

The authors declare that the research was conducted in the absence of any commercial or financial relationships that could be construed as a potential conflict of interest.
